# Inhibition of human esophageal squamous cell carcinomas by targeted silencing of tumor enhancer genes: an overview

**DOI:** 10.7497/j.issn.2095-3941.2014.02.002

**Published:** 2014-06

**Authors:** Jalil Pirayesh Islamian, Mohsen Mohammadi, Behzad Baradaran

**Affiliations:** ^1^Tabriz University of Medical Sciences, School of Medicine, Tabriz, East Asarbeidjan, Iran; ^2^Department of Radiation, School of Paramedicine, Shahid Beheshti University of Medical Sciences, Tehran, Iran; ^3^Immunology Research Center, Tabriz University of Medical Sciences, Tabriz, Iran

**Keywords:** Esophageal carcinoma, ionizing radiation (IR), oncogene, targeted therapy, siRNA

## Abstract

Esophageal cancer has been reported as the ninth most common malignancy and ranks as the sixth most frequent cause of death worldwide. Esophageal cancer treatment involves surgery, chemotherapy, radiation therapy, or combination therapy. Novel strategies are needed to boost the oncologic outcome. Recent advances in the molecular biology of esophageal cancer have documented the role of genetic alterations in tumorigenesis. Oncogenes serve a pivotal function in tumorigenesis. Targeted therapies are directed at the unique molecular signature of cancer cells for enhanced efficacy with low toxicity. RNA interference (RNAi) technology is a powerful tool for silencing endogenous or exogenous genes in mammalian cells. Related results have shown that targeting oncogenes with siRNAs, specifically the mRNA, effectively reduces tumor cell proliferation and induces apoptotic cell death. This article will briefly review studies on silencing tumor enhancer genes related to the induction of esophageal cancer.

## Introduction

Esophageal carcinoma, as a malignancy with poor prognosis, is the sixth leading cause of cancer-related death worldwide^1^. The incidence rate is close to the prevalence rate, indicating a short overall survival time^2^. Adenocarcinoma and squamous cell carcinoma (SCC) are the dominant histologies for esophageal carcinoma patients^3^. Evidence-based studies have suggested that genetic polymorphisms in carcinogen-metabolizing enzymes are important in determining an individual’s susceptibility to cancer^4^.

Management of esophageal carcinoma is based on the tumor extent according to the TNM classification and is divided into curative and palliative treatments. Patients with loco-regional disease (Stage I,II), in good medical condition, are often offered curative treatment.

Surgery remains the first choice for patients with early-stage cancers and is the standard against which all other treatment regimens are compared. Commonly used techniques for the resection of localized esophageal carcinoma are the transhiatal and right transthoracic approaches^5^. Although surgical techniques and postoperative care have improved, the reported mortality rates during operation remain as high as 4% to 10%^3^. Cisplatin in combination with 5-fluorouracil (5-FU) is considered the standard chemotherapy for esophageal carcinoma. The response rate for cisplatin, as a single agent, is approximately 20%^6^^,^^7^. The combination of cisplatin and continuous-infusion 5-FU has shown a response rate ranging from 35% to 65%^8^^,^^9^. Curatively intended radiation therapy can be performed as a conventional external radiotherapy, as intra-luminal brachytherapy, or in combination. However, the efficacy of conventional radiotherapy is limited by the following factors: (1) the presence of hypoxic, intrinsically radio-resistant, and repair-proficient tumor cells; (2) genetic, metabolic, and microenvironmental heterogeneity of tumors; and (3) undesirable damage to the normal healthy tissues^10^. Therefore, a significant improvement in therapeutic efficacy can be only achieved by developing effective approaches based on a comprehensive understanding of the radiobiology of tumor and normal tissues to enhance the radiation damage in tumors selectively while reducing the damage to normal tissues.

Adjuvant modality, a combination of chemotherapy and radiation therapy, is often used for down-staging of the tumor and for improving prognosis after surgery. Despite surgery or chemoradiotherapy, the prognosis of esophageal cancer treatment still remains poor, with a 5-year survival of approximately 10%. The failure of conventional therapy mainly occurs because tumors develop resistance to chemotherapy or radiation, and attempts to overcome resistance with higher doses of radiation and chemotherapeutics inevitably result in an unacceptable degree of toxicity and damage to normal tissues. The major limitation of all these treatments and their combinations is the lack of specificity for the tumor cell and toxicity to the patient^11^.

Neoadjuvant chemoradiotherapy for esophageal squamous cell carcinoma (ESCC) is beneficial in the setting of a complete pathological response. A gene expression study has shown that Rad51 expression is a useful predictive factor for the efficacy of neoadjuvant chemoradiotherapy in ESCC. Rad51 expression affects both chemo- and radio-sensitivity in various cancers^12^.

## Targeted therapy

Overall survival rates for ESCC patients remain substantially unchanged over the past several decades despite aggressive multimodality intervention^13^. However, recent insights into the epigenetic mechanisms associated with multi-step carcinogenesis^14^ and delineation of signal transduction pathways conferring chemo/radiation resistance in cancer cells^15^^-^^17^ provide new opportunities for the development of potentially effective targeted molecular therapies for ESCC and Barretts esophagus. Modern cancer therapies have evolved from non-specific cytotoxic agents that affect both normal and cancer cells to targeted therapies and personalized medicine.

Targeted therapies are directed at the unique molecular signatures of cancer cells to achieve significant efficacy with low toxicity^18^. Molecular studies of human esophageal tumors have revealed frequent genetic abnormalities (**Table 1**)^4^. Regardless of patient origin and suspected etiological factors, genetic changes that are consistently observed in ESCC are as follows: (1) alterations in tumor suppressor genes, specifically p53, resulting in altered DNA replication and repair, cell proliferation, and apoptosis; (2) disruption of the G_1_/S cell cycle checkpoint and loss of cell cycle control; and (3) alterations in oncogene function resulting in deregulation of cell signaling cascades^19^^,^^20^. In a recent comprehensive genomic analysis of 158 ESCC cases, as part of the International Cancer Genome Consortium research project, whole-genome sequencing was applied to 17 ESCC cases and whole-exome sequencing to 71 cases, of which 53 cases plus an additional 70 ESCC cases were unused in whole-genome and whole-exome sequencing, were subjected to array comparative genomic hybridization analysis. Eight significantly mutated genes were identified, among of which six genes are well-known tumor-associated genes (*TP53, RB1, CDKN2A, PIK3CA, NOTCH1,* and *NFE2L2*), whereas two have not previously been described in ESCC (*ADAM29* and *FAM135B*)^21^. In particular, *FAM135B* is identified as a novel cancer-implicated gene that promotes the malignancy of ESCC cells. In addition, a correlation between CD133 expression and the immunolocalization of several markers, such as p53, p16, p27, murine double minute 2 (MDM2), Ki-67, and epidermal growth factor receptor (EGFR), was observed. These indicators are known as prognostic markers or tumor proliferation factors in ESCC^22^. In a study by Nam *et al*.^23^, positive expressions of p53, Rb tumor suppressor protein (pRb), hMLH1, and MDM2 were observed in 40%, 46.7%, 40%, and 66.7% of the tissue specimens, respectively.

**Table 1:** Molecular alterations in human esophageal squamous cell carcinoma^4^

Loss of heterozygosity	1p, 3p, 4, 5q, 9, 11q, 13q, 17q, 18q
Loss of tumor suppressor gene function	p53 mutation
Methylation and/or loss of p16MST1 and or p15	Reduced Rb expression
Gene amplification	cyclin D1
	HST-1
	EGFR
	INT-2
Increased expression	iNOS
	hTERT
	BMP-6
	COX-2
	c-myc
	β-catenin

Other genetic alterations that are commonly associated with clinical tumors include *p53* mutations^24^^,^^25^; loss of *p16MST1* and/or *p15*^26^, and/or *RARβ* expression^27^; amplification of *cyclin D1*, *HST-1*, *EGFR* and *INT-2*^28^^-^^31^; and elevations in *iNOS, hTERT, BMP-6, COX-2* and *c-Myc* expression^32^^-^^36^; as well as cytoplasmic *β-catenin* levels^37^. One or several of these alterations contribute to the growth and metastatic potential of these tumors.

Two other putative tumor suppressor genes, namely, *FEZ-1* on chromosome 8q22 and *DLC1* on 3p21, have been identified as novel candidates that may serve a function in esophageal carcinogenesis, given that their expressions are absent in some sporadic tumors^38^^,^^39^. In contrast to the extensive literature on genetic alterations in frank tumors, limited information is available on genetic alterations in precancerous lesions of the esophagus.

The *p53* gene results in cell cycle arrest through p21WAF1 induction, which sequesters CDKs by down-regulating bcl-2 (known as a key molecule in the regulation of apoptosis or programmed cell death), while up-regulating bax^40^. This process induces apoptosis^18^. MDM2, also known as HDM2 in humans, is a negative regulator of the tumor suppressor p53^18^. MDM2 belongs to a large family of ring-finger-containing proteins and functions mainly, if not exclusively, as an E3 ligase^41^^,^^42^. MDM2 targets p53 for mono- and/or poly-ubiquitylation, thereby controlling its localization and/or levels through proteasome-dependent degradation. MDM2-mediated mono-ubiquitylation of p53 results in cytoplasmic sequestration, whereas poly-ubiquitylation triggers p53 degradation. MDM2 also suppresses p53 function by binding to p53, thereby hindering its capability to interact with the basal transcriptional machinery and transcriptional co-activators, such as p300^42^^,^^43^. In response to DNA damage, phosphorylation of p53 on Ser20 and of MDM2 on Ser395, as mediated by kinases such as ATM, interrupts the p53-MDM2 interaction, thus resulting in the nuclear accumulation of p53 and the activation of its transcriptional program^44^. MDM2 is overexpressed in a various human cancers, including melanoma, non-small cell lung cancer, breast cancer, esophageal cancer, leukemia, non-Hodgkin’s lymphoma, and sarcoma^18^. The tumor suppressor p53 is a powerful anti-tumor molecule that is frequently inactivated by mutations or deletions in cancer. However, half of the human tumors express wild type (wt) p53, and its activation by antagonizing its negative regulator MDM2 might offer a new therapeutic strategy^45^. Proof-of-concept experiments have demonstrated the feasibility of this approach *in vitro*, but the development of pharmacological inhibitors remains a challenge. Potent and selective small-molecule MDM2 inhibitors have recently been identified^46^^,^^47^. Studies of these compounds have strengthened the concept that selective and non-genotoxic p53 activation is a viable alternative to current cytotoxic chemotherapy. However, clinical validation remains pending. As a single agent, Nutlin-3a only increases apoptosis and decreases survival preferentially in wt p53-expressing cells. Although the present data support the notion that initiating the growth suppressive and pro-apoptotic activity of p53 by MDM2 antagonists is a potentially valuable strategy for treating OS with wt p53, further studies are needed to reveal the true therapeutic potential of this approach^48^.

RNA interference (RNAi) is the process of sequence-specific, post-transcriptional gene silencing directed by short interfering 21-23 nucleotides (nt) double-stranded RNA (siRNA). A number of studies have demonstrated that the introduction of siRNAs into mammalian and human cells causes specific and effective suppression of the corresponding mRNA molecules. siRNAs can inhibit the *in vivo* expression of endogenous genes, providing further support to the notion that oncogene-specific siRNAs may be new alternatives for gene-specific therapeutics of human cancers. For example, MDM2 targets p53 protein for degradation in the ubiquitin pathway, resulting in the abrogation of its antiproliferative and apoptosis-promoting effects. In addition, studies have shown that p14 induces the degradation of the proto-oncogene MDM2, which destabilizes p53. Furthermore, cell-cycle arrest mediated by p14 can be terminated in cells lacking functional p53, indicating that p14 may act upstream of p53. Significant alterations in the chromatin structure occur during multistep esophageal carcinogenesis^49^. Global DNA demethylation results in de-repression and activation of an operator gene through the deactivation of a repressor gene of imprinted alleles, such as H19 and IGF2^50^^,^^51^, as well as the up-regulation of germ cell-restricted genes, many of which are linked to the X chromosome and encode proteins that are recognized by tumor reactive lymphocytes^52^^-^^54^. Paradoxically, site-specific DNA methylation silences a variety of tumor suppressor genes, including *p16*, *RASSF1A*, *FHIT*, *E-cadherin*, and *RARb* in esophageal cancers^55^^-^^58^. Hasan *et al*.^59^ also showed that TC21 knockdown sensitizes ESCC to cisplatin. Several of these tumor suppressor genes are methylated in Barretts epithelium, indicating that epigenetic events that perturb cell cycle regulation occur extremely early during esophageal adenocarcinogenesis^55^^,^^60^. By contrast, the effects of CTA expression regarding the malignant phenotype of esophageal cancers have not been clearly established^61^^,^^62^. The implications of the methylation-mediated inactivation of tumor suppressor genes are evident. For example, restoration of p16 or FHIT expression by gene transfer techniques mediates growth arrest and apoptosis in cultured esophageal cancer cells^63^^,^^64^. Furthermore, several studies suggest that the aberrant methylation of tumor suppressor genes coincides with adverse response to therapy in esophageal cancer patients^56^^,^^65^^,^^66^. Recent studies suggest that the aberrant activity of DNA methyltransferases and histone deacetylases (HDACs) may contribute to the inactivation of tumor suppressor gene expression and perturbed cell cycle regulation in aerodigestive tract malignancies^67^^,^^68^. In contrast to genes that have been mutated or deleted, the expression of epigenetically silenced tumor suppressors can be restored in cancer cells by pharmacologic compounds, including DNA demethylating agents and HDAC inhibitors^69^. Although HDAC inhibitors, such as FK228, modulate the chromatin structure through the acetylation of the core histone proteins, these agents also disrupt oncoprotein signaling by mechanisms that are remarkably analogous to those mediated by geldanamycin derivatives^70^^,^^71^. Indeed, FK228-mediated growth arrest in cancer cells coincides with signaling inhibition through the EGFr-ras-raf-Erk and P13K/AKT pathways^70^. Furthermore, FK228 and other HDAC inhibitors markedly enhance p21expression^63^^,^^70^. Abrogation of p21 expression by flavopiridol enhances depsipeptide-mediated apoptosis in malignant pleural mesothelioma cells, thus contributing to cell cycle arrest, but also inhibiting apoptosis of cancer cells following exposure to a variety of conventional chemotherapeutic agents and novel antitumor compounds^72^.

## Discussion

Surgery, radiation therapy, and chemotherapy have been the main modes of treatment for human malignancies for more than 40 years. The use of a combination of radiation and chemotherapy is often called chemoradiation in the medical literature^73^. The rate of tumor growth and expansion is controlled by the balance between cell proliferation/survival and apoptosis, which are strictly regulated processes in normal cells. However, apoptosis is disrupted in cancer cells and tumors, and the cell cycle proteins required for cell survival and proliferation are up-regulated or are continuously activated^74^. In recent years, considerable insight into the mechanisms of aerodigestive tract carcinogenesis has been gained. The aforementioned studies indicate that the manipulation of gene expression as a means to restore cell cycle regulation and induce apoptosis is feasible in esophageal cancer cells *in vitro*, as well as in clinical settings. Furthermore, recent laboratory experiments have demonstrated that novel compounds that inhibit survival signaling markedly enhance the efficacy of conventional therapeutic regimens for esophageal cancer. These data clearly support the evaluation of these combination treatment regimens using well-designed clinical protocols. We are entering a new era as regards the treatment and prevention of cancer, but whether the targeted molecular therapies can inhibit the pathogenesis and clinical progression of esophageal malignancies remains to be proven^75^.

Curative treatment of malignant tumors with ionizing radiation (IR) was introduced 80 years ago^76^. The conditions of the tumor microenvironment that favor tumor cell survival after IR include hypoxia and secretion of radiation-protective cytokines and growth factors that promote the growth and survival of tumor tissues. Tumor radioresistance serves an important function in treatment failure for esophageal cancer. Therefore, the mechanisms involved in tumor radioresistance must be determined to improve prognosis^77^. Moreover, a number of genes have been implicated in the response mechanism of eukaryotic cells to IR. These genes include a number of cell cycle, checkpoint, and DNA repair genes, as well as mediators of apoptosis, such as p53, bax, and Bcl-2. The expression or repression of related genes is associated with cell survival or cell death in simple model systems, but shed no light on intercellular events in in-situ tumors or in the clinical outcome of radiotherapy. In addition, studies have shown that miRNA expression fingerprints correlate with the clinical and biological characteristics of tumors, including tissue type, differentiation, aggression, response to therapy, and prognosis^78^. For example, analysis of MDM2 overexpression in relation to *p53* gene status has revealed significant associations between *p53* missense mutations and the lack of detectable MDM2 protein expression^79^. Inhibition of MDM2 can restore p53 activity in cancers with wt p53, resulting in anti-tumor effects with apoptosis and growth inhibition^18^. The silencing of HDM2 mRNA directly enhances MCF-7 cell apoptosis and decreases cell proliferation. These results provide strong evidence that the siRNA technology can be an effective method for inhibiting oncogene expression and activating apoptotic and tumor suppressor genes^80^. MDM2 is a critical component of the responses to both ionizing and UV radiation^81^. The decreased levels of MDM2 sensitize cells upon IR. Thus, MDM2 is a potential target for therapeutic intervention because its inhibition may radiosensitize the subset of human tumors expressing wt p53, making radiotherapy more effective^81^.

Non-specific cytotoxic agents that affect both normal and cancer cells in targeted therapies and personalized medicine are considered as novel cancer therapeutics. Targeted therapies are directed at the unique molecular signatures of cancer cells for enhanced efficacy with low toxicity^18^. Cancer is a multistep genetic and epigenetic disease with a complex etiology, and cancer cells have been characterized by several defects, such as mutations, down-regulation, over-expression, and deletions of oncogenes and tumor suppressor genes^74^. Expression array technology has become an important method for many applications, including the identification of disease-related and treatment-responsive genes, as well as the determination of carcinogenicity, toxicity, and safety of drugs^82^. These techniques have the capability to identify genes with expressions that correlate with ESCC because these genes could be potential candidates as molecular markers for the prevention and early detection of ESCC. Moreover, identification of genes that are differentially expressed between radiosensitive and radioresistant cancer cells is important for predicting the clinical effectiveness of radiotherapy^83^. Therefore, activation of the cellular apoptotic program is a current strategy for the treatment of human cancers. Studies have demonstrated that radiation and standard chemotherapeutic drugs kill some tumor cells through the induction of apoptosis^84^. Most chemotherapeutic agents and radiation therapy target the DNA. Non-DNA targets may be effective in killing the cell or modifying the cell in such a way that it becomes more susceptible to cell killing after radiation-induced damage^85^. Recent advances in the molecular biology of esophageal cancer have documented the function of genetic alterations in tumorigenesis and have facilitated the development of potential new therapeutic approaches designed to target such genetic alterations^11^. Further study is needed to elucidate the markers or combinations of markers that best enhance the effects of radiotherapy in ESCC. Such markers might prove valuable, not only as clinical predictors, but also as targets for ESCC treatment. For example, the treatment might result in an increased sensitivity if these abnormal functions and expressions return to normal^86^.

The siRNA complexes silence gene expression *in vitro* or *in vivo* with excellent specificity in cells bearing the receptor recognized by the antibody and can target cells, such as primary lymphocytes, which are refractory to lipid-mediated transfection. RNAi possesses high specificity and high efficiency in down-regulating gene expressions, making it a potential therapeutic strategy against human cancer. Several molecules involved in cell-cycle regulation have been targeted for RNAi intervention in an effort to suppress cancer cell growth. Two cell-cycle regulators, namely, pRb and p53, are of special importance in cancer therapy and worthy of discussion^87^. RNAi has facilitated the identification and study of the components of apoptosis and survival pathways, thus enabling the identification of specific gene targets for improving the effectiveness of cancer therapies.

## Conclusion

Significant advances in understanding the molecular biology of esophageal cancer have resulted in the application of gene therapeutic methods, in which genetic material is transferred into human cells and expressed in those cells for a therapeutic purpose. Many of the genes described above may contribute to the resistance to chemotherapy or irradiation. For example, the expression of antiapoptotic proteins by cancer cells is an important mechanism by which cancer cells resist chemotherapy or irradiation. Using RNAi to target antiapoptotic proteins may be a promising strategy for use in conjunction with chemotherapy and radiotherapy for cancer treatment. RNAi therapy can potentially be used in conjunction with chemotherapy, radiotherapy, and/or immunotherapy. The same types of tumors, even with similar clinical phases, differ in terms of radiosensitivity. In recent years, many genes related to the radiosensitivity of tumor cells have been found. Studies focus on controlling the radiosensitive genes and adjusting the fraction dose and interval of radiation, as well as on how to realize the individualization of radiotherapy, change tumor cell radiosensitivity, and reduce the normal tissue damage to achieve the optimum therapeutic effects. Safety and effectiveness are the key factors in gene therapy, and its control mechanism affects its targets. siRNA technology has several major advantages over other post-transcriptional gene silencing techniques, such as the antisense and gene knockout technologies. siRNA technology is easier to deliver, requires only small doses of siRNA to produce its silencing effect, and can directly inactivate a gene at almost any stage in development with biological molecules through a number of theoretically possible reactions. The gene therapy in the field of esophageal cancer is in the primitive stage compared with those in lung, head and neck, as well as brain cancers.
